# Supplementary material for: Novel Motion Sequences in Plant-Inspired Robotics: Combining Inspirations from Snap-Trapping in Two Plant Species into an Artificial Venus Flytrap Demonstrator
Source: Biomimetics (Basel). 2022 Jul 22;7(3):99. doi: 10.3390/biomimetics7030099 (PMC9330566; doi:10.3390/biomimetics7030099)
Supplement: Supplementary file 1 [file biomimetics-07-00099-s001.zip › biomimetics-1796118-supplementary.pdf]

biomimetics

Supplementary Materials

Supplementary Materials: Novel Motion Sequences in Plant-Inspired Robotics: Combining Inspirations from Snap-Trapping in Two Plant Species into an Artificial Venus Flytrap Demonstrator

Falk J. Tauber [1,2,†,*], Philipp Auth [1,†], Joscha Teichmann [1], Frank D. Scherag [2,3] and Thomas Speck [1,2,4]

[1] Plant Biomechanics Group, Botanic Garden, University of Freiburg, 79110 Freiburg, Germany; ph.auth@gmx.de (P.A.); joscha.teichmann@studmail.w-hs.de (J.T.); thomas.speck@biologie.uni-freiburg.de (T.S.)
[2] Cluster of Excellence livMatS @ FIT-Freiburg Center for Interactive Materials and Bioinspired Technologies, University of Freiburg, 79110 Freiburg Germany; scherag@imtek.de
[3] Laboratory for Chemistry and Physics of Interfaces CPI, Department of Microsystems Engineering-IMTEK, University of Freiburg, 79110 Freiburg Germany
[4] Freiburg Materials Research Center (FMF), University of Freiburg, 79110 Freiburg Germany
* Correspondence: falk.tauber@biologie.uni-freiburg.de; Tel.: +49-761-203-95152
† Authors contribute equally to this work

Citation: Tauber, F.J.; Auth, P.; Teichmann, J.; Scherag, F.D.; Speck, T. Novel Motion Sequences in Plant-Inspired Robotics: Combining Inspirations from Snap-Trapping in Two Plant Species into an Artificial Venus Flytrap Demonstrator. *Biomimetics* **2022**, *7*, 99. https://doi.org/10.3390/biomimetics7030099

Academic Editor: Jinyou Shao

Received: 15 June 2022
Accepted: 15 July 2022
Published: 22 July 2022

Publisher's Note: MDPI stays neutral with regard to jurisdictional claims in published maps and institutional affiliations.

Copyright: © 2022 by the authors. Submitted for possible open access publication under the terms and conditions of the Creative Commons Attribution (CC BY) license (https://creativecommons.org/licenses/by/4.0/).

1. Introduction

Within this supplementary material document, we describe in detail the manufacturing process of the compliant foil based VFf demonstrators and show supplementary data of their characterization.

2. Materials and Methods

2.1 Standardized production of the compliant foil demonstrators

The base unit of the compliant foil based demonstrator VFf systems consist of a PET foil and a flexible microscopic slide. The manufacturing process is described in detail below.

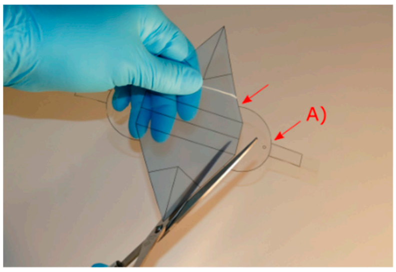

1. The demonstrator is cut out. Sharp edges should be avoided. Special attention I advised at edge A). It must be rounded to increase the demonstrator's lifespan.

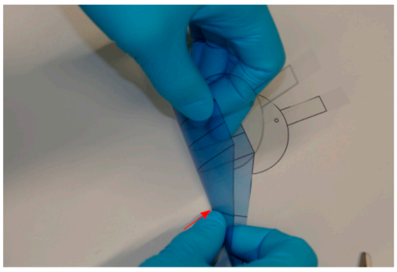

2. The lobes are folded. The bend should be until the marked spot (the edge of the two triangles).

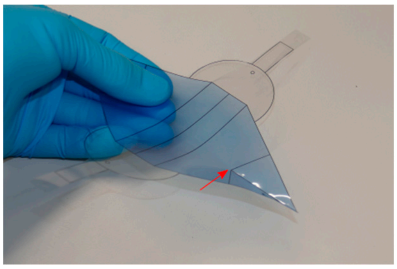

3. Once the lobes are folded, a slight curvature is already visible

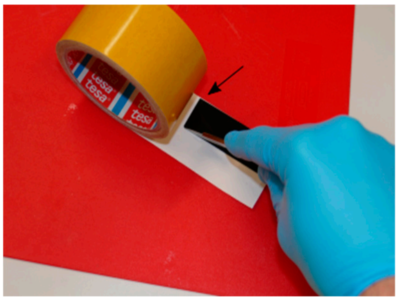

4. The plastic backbone is placed on the double-sided tape. Then it is cut out precisely using a scalpel on a cutting board.

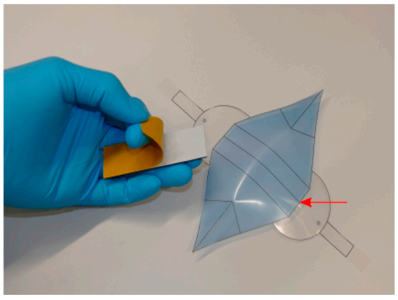

5. The protective foil is removed. The Backbone is carefully placed on the edge of the demonstrator, aligning the backbone with the printed mark on the foil.

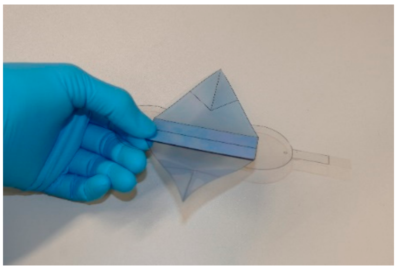

6. The backbone should be pressed firmly onto the foil. The backbone is facing downwards.

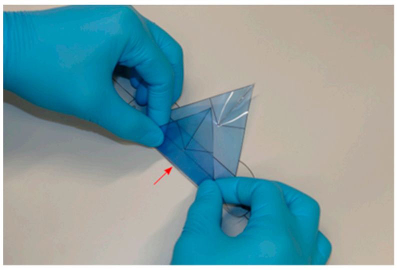

7. The lobes are folded upwards along the plastic backbone. The spatial curvature should be visible. Both sides should be folded equally.

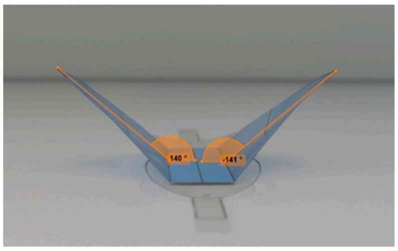

8. An angle of approx. 140° between backbone and the lobes resulted in proper functionality.

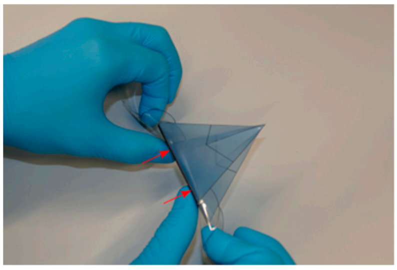

9. The closing mechanism is triggered by applying force on the backbone at both sides while holding the round structures on the sides of the foil.

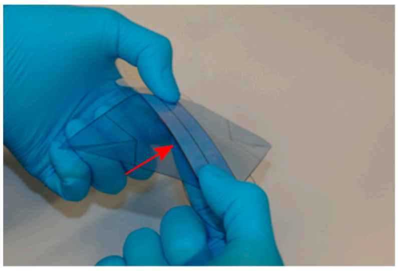

10. The opening mechanism is triggered by applying force to the center of the bottom side of the of the backbone. Counterforce is applied on the upper side on both sides.

2.2. Kinematic analysis setup:

Kinematic tracking was performed with the Kinovea 0.9.1, to achieve a sufficient automated tracking the lobes tips were blackened and a white background was used. For sufficient lighting the highspeed video chamber was outfitted with two constant light sources (Brennenstuhl H500 or Veritas Constellation 120E).

3. Kinematic analysis of the biological role model

Table S1. Kinematic analysis of the Dionaea muscipula was performed with the same setup as the magnetic and pneumatic demonstrator.

Cycle	Plant	Angle [°]	Time [ms]	v_{max_left}	v_{max_right}	Opens first	Width	Length
1	P1F1	77	158	0,0981	0,0794	same	25,67	11,06
1	P1F2	89	682	0,075	0,064	right	27,07	9,82
1	P1F3	93	165	0,18	0,23	left	26,26	10,56
1	P2F1	72	160	0,06	0,081	same	23,22	9,88
1	P2F2	93	479	0,051	0,042	right	27,32	12,83
1	P3F1	95	223	0,082	0,074	left	20,11	9,82
1	P3F2	86	217	0,086	0,064	same	21,82	9,29
2	P1F1	83	1529	0,0190	0,0164	same	29,26	12,04
2	P1F2	91	148	0,1006	0,0682	same	22,45	11,51
2	P1F3	66	211	0,0516	0,0634	left	23,42	11,81
2	P1F4	95	533	0,1975	0,2449	right	28,48	12,19
2	P1F5	98	922	0,1147	0,2042	left	24,93	9,63
2	P2F1	89	825	0,0811	0,0680	right	25,61	11,51
2	P2F2	73	297	0,0700	0,0786	right	24,35	10,06
2	P2F3	83	252	0,0387	0,0467	right	23,38	9,96
3	P1F1	89	420	0,078638	0,07863837	same	25,11	9,85
3	P1F2	94	1821	0,12475	0,09156295	right	26,21	10,45
3	P1F3	78	223	0,101229	0,0506049	same	24,65	9,51

3	P1F4	97	1124	0,175376	0,1197035	right	26,62	13,04
3	P1F6	87	480	0,127019	0,1087012	right	24,56	12,32
3	P2F1	71	778	0,027051	0,0333718	same	27,36	14,11
3	P2F2	87	332	0,064529	0,05722146	same	23,98	9,8
3	P2F3	83	293	0,03705	0,05147836	same	24,43	9,43
1	P4F1	91	1730	0,105819	0,05207146	right	28,98	13,25
1	P4F2	100	887	0,109335	0,1977565	left	26,81	10,02
1	P5F1	92	228	0,132798	0,09611847	same	27,57	9,55
1	P5F2	77	1137	0,038627	0,02813612	left	25,14	11,55
1	P5F3	83	522	0,06374	0,05593698	same	27,88	12,23

4. Kinetic energy measurements

Each demonstrator required a specific testing setup to apply force according to the actuation scenario.The pneumatic AVF had two force application cases, first the force application of the outer cushions closing the lobes and second force of the central cushion opening the lobes again. The pneumatic AVF was fixed in an upside-down configuration to the setup and the cushion casing was kept open. With a modular pressure stamp it was possible to apply forces in correspondence to the two described cases. In case of the magnetic demonstrator, force was applied directly onto the magnets. The force generated by the SMA springs was measured. The springs were stretched to their initial lengths of 115 mm and then clamped into the testing machine. A temperature chamber was used to heat the surrounding air which led to the springs attempting to contract. As contraction was prevented by the testing machine, no movement occurred but force was generated.

Table S2. Maximum force generated by SMA springs at 65°C.

Name	F_{max} [N]
Spring1.1	6,77
Spring1.2	7,86
Spring1.3	6,64
Spring 1.4	6,89
Spring 1.5	6,57
Spring 1.6	6,56
Spring 1.7	6,53
Spring 2.1	4,96
Spring 2.2	4,87
Spring 2.3	4,45
Spring 2.4	4,51
Spring 2.5	3,99
Spring 2.6	4,65
Spring 2.7	4,66
Spring 3.1	4,16

Spring 3.2	4,40
Spring 3.3	4,26
Spring 3.4	4,36
Spring 3.5	4,18
Spring 3.6	4,01
Spring 3.7	3,82

5. Motion analysis of discrete repetitive motion generation by pneumatic actuation

The pneumatic demonstrator was actuated using a specific pneumatic test bench Figure S1. The function of the test bench is described in detail in another publication [25]Klicken oder tippen Sie hier, um Text einzugeben.. For our purposes the software of the actuation pattern was modified to fit the desired actuation squence.

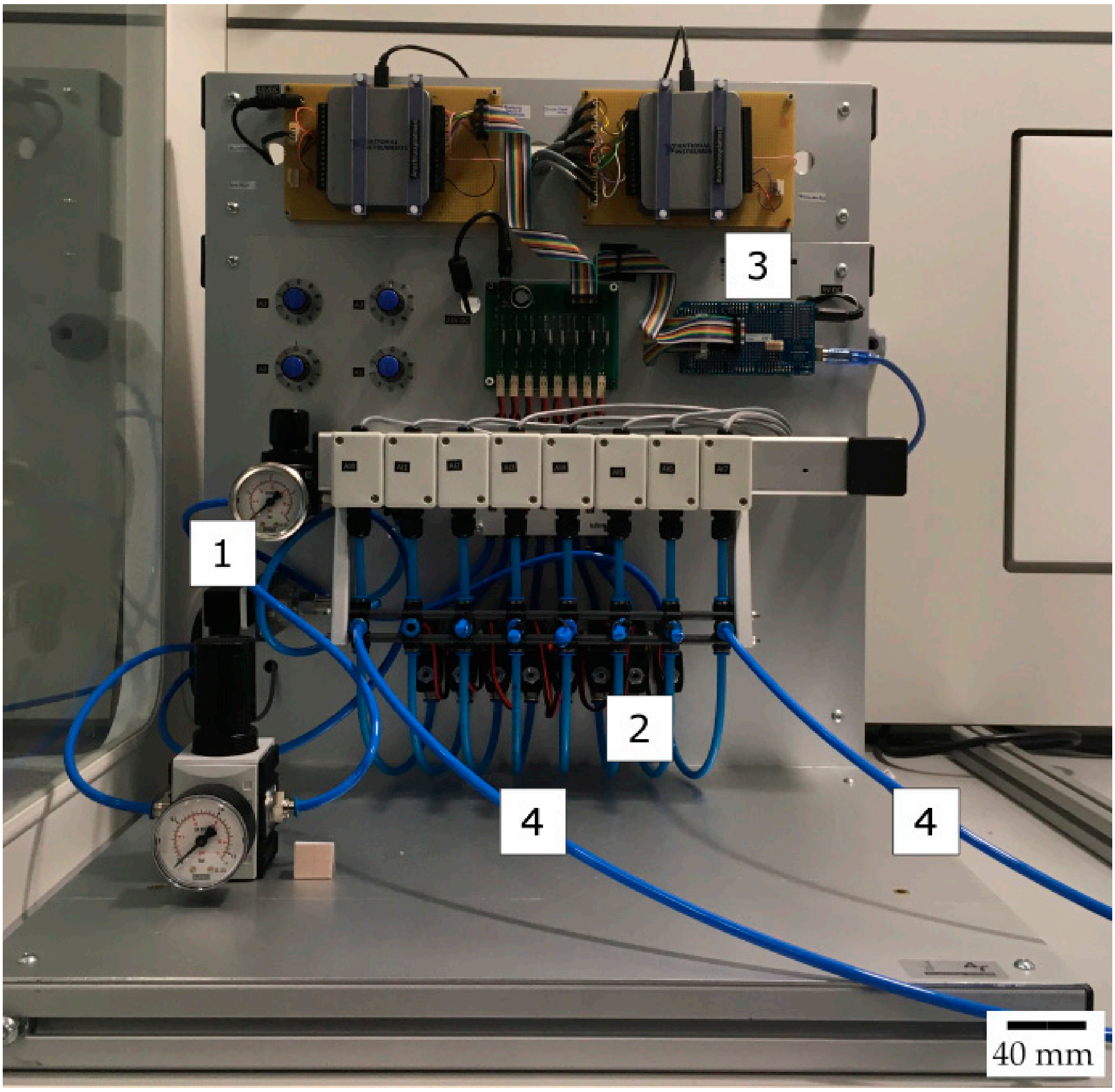

Figure S1. The pneumatic test bench provided pressurized air for the demonstrator. Pressurized air from the laboratory hood is reduced to desired pressure (1). The Arduino (2) controls the opening and closing of the magnet valves (3) that regulate airflow (4) to the demonstrator.

Table S3. The actuation pattern, actuation time and actuation pressure that were used to achieve repetitive actuation are displayed.

Actuation pattern (O CLOSED; X OPEN)	Pressure outside cushions (combined)	Pressure central cushion	Magnet valve opening time	Total actuation time in one cycle	Duration of one complete cycle
Side: 00XXX000 Middle: 0000XXX0	0.8 bar	0.8 bar	40 ms	200 ms	8*40 ms = 320 ms
Side: 00XXX000 Middle: 0000XX00	0.8 bar	0.8 bar	75 ms	300 ms	8*75 ms = 600 ms
Side: 00XXX000 Middle: 0000XX00	0.8 bar	0.8 bar	100 ms	400 ms	8*100 ms = 800 ms

6. Motion analysis of contactless actuation of the demonstrator by a rotating magnetic field

For actuation of the magnetic demonstrator two round neodymium magnets (15x2 mm, 30 g per unit) were glued onto one side of the demonstrator using double sided tape. The magnetic actuator was mounted at a fixed position as displayed in Figure S2. The magnetic stirrer (IKA RCT B 5000) was turned on at a rotating frequency of 200 rpm. The frequency was raised in increments of 100 rpm up to 1500 rpm giving 60 seconds of adjusting time each step before recording a video. 18 videos were recorded at a framerate of 1000 fps and contained 3000 frames each. One camera was aligned with the demonstrator, the other one was placed perpendicularly to the plane of motion. The videos were recorded with a software (StreamPix, NorPix Inc.) that enables simultaneous, synchronized recording with two cameras. Three different demonstrators were tested using the same setup.

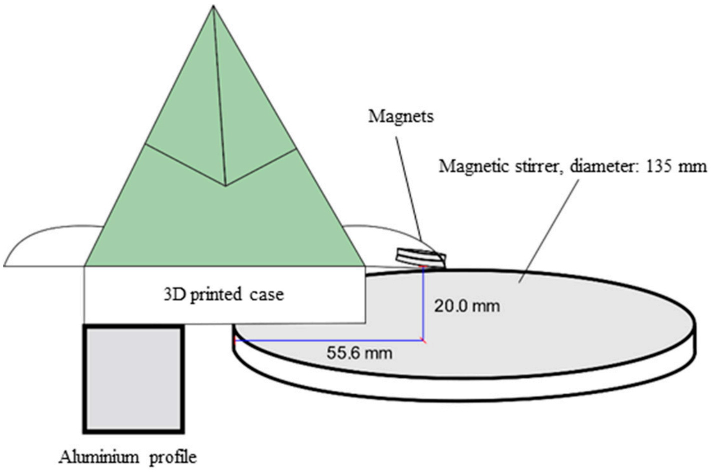

Figure S2. Setup for magnetic demonstrator testing. All magnetic demonstrators are tested using the same setup. Therefore, distances between the magnet and the stirrer are kept equal.

The flapping frequency was measured via video analysis. A corresponding increase of flapping frequency and motion was observed with increasing rotations per minute (rpm) of the magnetic stirrer. See Figure S3 and Table S4 for the corresponding data.

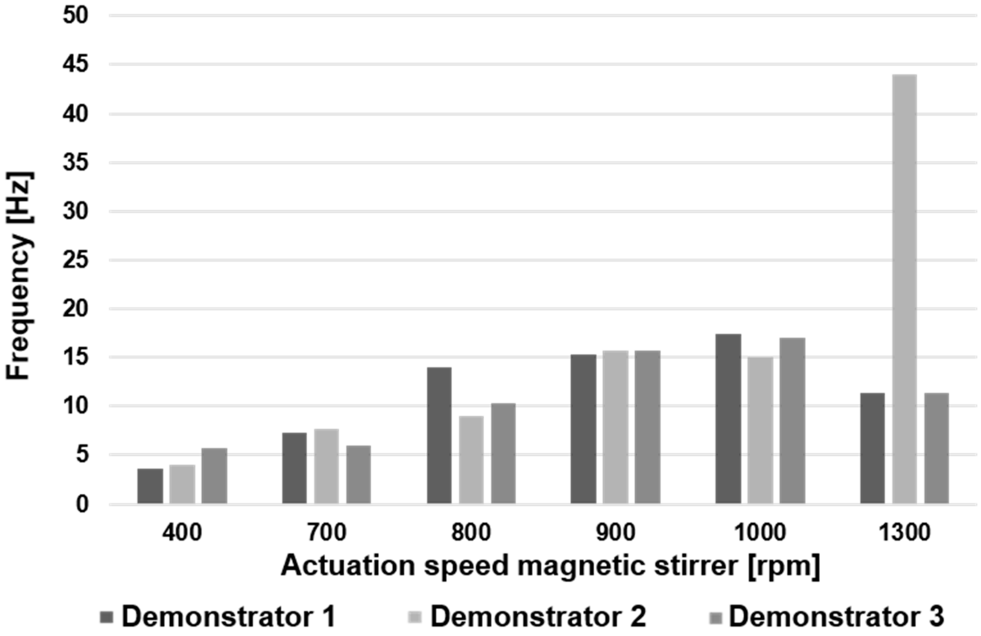

Figure S3. Frequency measurements of magnetic demonstrators. Flapping frequency of the magnetic demonstrators in correspondence to changing actuation speed.

Table S4. Flapping frequency corresponding to different actuation speeds of the magnetic demonstrators.

Demonstrator	Flapping frequency at 400 rpm [Hz]	Flapping frequency at 700 rpm [Hz]	Flapping frequency at 800 rpm [Hz]	Flapping frequency at 900 rpm [Hz]	Flapping frequency at 1000 rpm [Hz]	Flapping frequency at 1300 rpm [Hz]
1	3,67	7,33	14	15,33	17,33	11,33
2	4	7,67	9	15,67	15	44
3	5,67	6	10,3	15,67	17	11,33

Raw data for the speed kinematic analysis exceeds the capacity of this document. Upon reasonable request data is available.

7. Environmentally triggerable systems: Thermally driven AVF using shape memory ally (SMA) springs

Five SMA springs were tested to determine the critical temperature. For further testing three springs were chosen that showed similar cpontraction behaviour. Spring 4 starts to contract at 56°C whereas the other springs begin to contract at 62-64°C Figure S4. Spring three contracted slower compared to the other springs. Therefore, springs 1, 2 and 5 were picked for further testing.

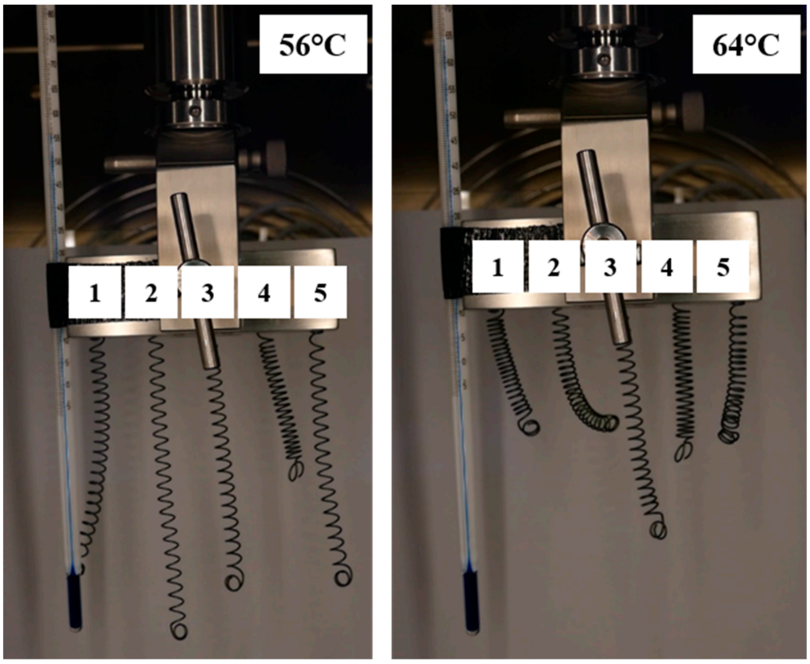

Figure S4. Determination of critical temperature and contraction behavior of the five SMA-springs.

The SMA springs were measured at two different temperatures to determine to change of length and the critical temperature.

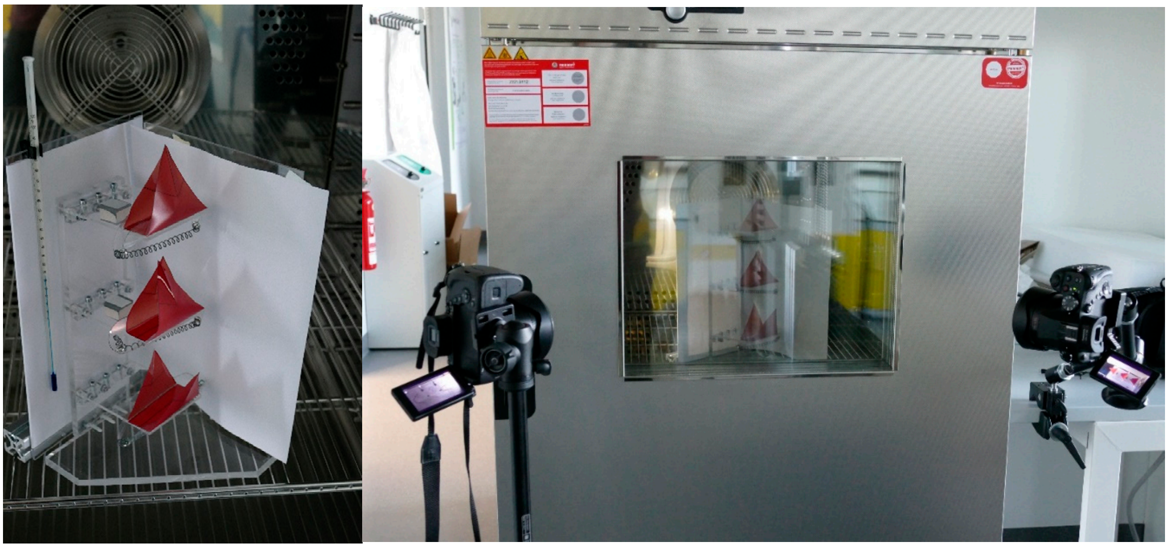

Figure S5. Climate chamber setup for SMA-spring demonstrator measurements. Three VFs with attached SMA springs were tested simultaneously (left). They were mounted on a plexiglass shelf and placed in a climate chamber (Memmert CTC 265) (right).

During the measurements with the climate chamber thermal stratification was observed. Ensuring that each demonstrator reached the transition temperature, three temperature sensors were fixed one each at the level of the demonstrators, to record and control the temperature surrounding the SMA springs. In Figure S 5 an exemplary heat distribution graph is shown.

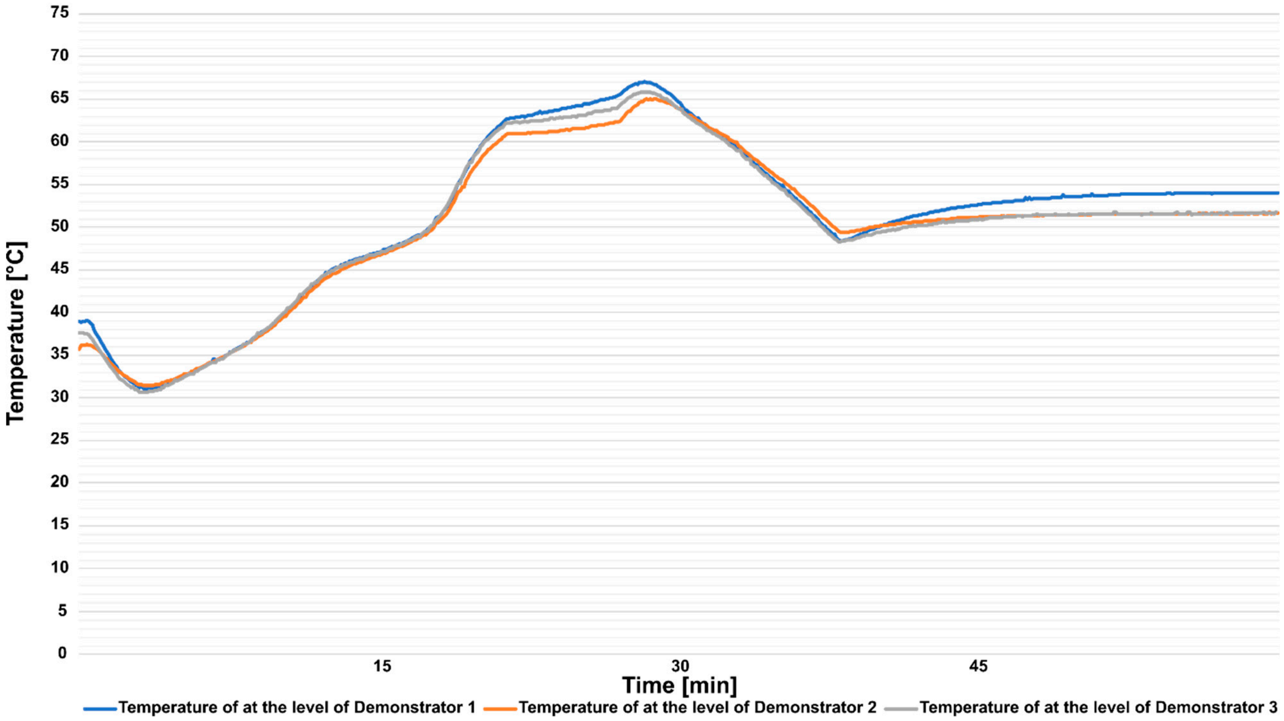

Figure S6. Temperature distribution inside the climate chamber at the different levels of the demonstrators.

Table S5. Length change of SMA springs at different temperatures when mounted to a foil demonstrator. The experiment was performed using the climate chamber. T1 = starting temperature, T2 = end temperature, L1.X = spring length of spring X at starting temperature, L2.X = spring length of spring X at end temperature, deltaX = difference in spring length at starting and end temperature.

T1 [°C]	T2 [°C]	L1.1 [cm]	L1.2 [cm]	L1.3 [cm]	L2.1 [cm]	L2.2 [cm]	L2.3 [cm]	delta1 [cm]	delta2 [cm]	delta3 [cm]
51	55	12,9	13,7	12,44	12,9	13,54	12,23	0	0,16	0,21
51	55	11,96	13,1	12,09	11,91	13,1	11,87	0,05	0	0,22
50	55	11,94	13,08	12,09	11,74	13	11,85	0,2	0,08	0,24
50	55	11,54	12,22	11,46	11,62	12,18	11,43	-0,08	0,04	0,03
50	55	11,65	12,61	11,56	11,54	12,44	11,54	0,11	0,17	0,02
50	55	11,4	12,11	11,32	11,4	12,11	11,28	0	0	0,04
50	55	11,19	11,97	11,27	11,07	11,8	11,16	0,12	0,17	0,11
55	60	11,25	11,76	11,17	9,54	11,74	11,04	1,71	0,02	0,13
55	60	11,18	11,84	11,17	8,62	12,15	11,14	2,56	-0,31	0,03
55	60	11,4	12,24	10,96	8,42	11,81	11,14	2,98	0,43	-0,18
55	60	11,54	12,16	11,33	10,14	11,94	11,43	1,4	0,22	-0,1
55	60	11	11,96	11,33	9,73	11,88	11,29	1,27	0,08	0,04
55	60	11,05	11,7	10,93	10,4	11,6	10,96	0,65	0,1	-0,03
55	60	11,2	12,1	11,21	8,87	12,08	11,45	2,33	0,02	-0,24
55	65	11,45	12,1	11,59	6,01	8,06	8,43	5,44	4,04	3,16
55	65	10,73	11,34	10,66	5,82	7,87	9,86	4,91	3,47	0,8
55	65	11	11,87	11,49	6,42	8,37	10,65	4,58	3,5	0,84
55	63	11,55	12,06	11,54	6,82	8,68	10,36	4,73	3,38	1,18
55	65	10,68	11,46	11,24	6,61	8,21	10,5	4,07	3,25	0,74
55	65	11,38	11,78	11,43	6,94	8,58	10,62	4,44	3,2	0,81
55	65	11,34	11,92	11,37	7,14	8,73	10,81	4,2	3,19	0,56

Table S6. Closing times of the SMA spring demonstrator at different temperatures.

Temperature [°C]	Closing time [s] Spring 1	Closing time [s] Spring 2	Closing time [s] Spring 3
55			
55			
55			
55			
55			
55			
55			
60	04:12		
60	04:36		
60	01:40		
60	03:04		

60	06:45		
60	03:54		
60	04:06		
65	01:56	02:06	04:08
65	01:05	00:36	07:09
65	03:41	04:28	04:04
65	02:58	03:05	07:07
65	04:26	05:05	06:32
65	04:04	03:11	04:37
65	03:59	03:28	03:29

8. Environmentally triggerable systems: Stimulus combination humidity and temperature

8.1 Hydrogel application:

A hydrophilic terpolymer was synthesized by free radical copolymerization of the three monomers N,N-Dimethyacrylamide (DMAA, 92.5 mol %), 4-Methacryloyloxybenzophenone (MABP, 5 mol %) and Na-4-styrenesulfonate (SSNa, 2.5 mol %). An amount of 300 µl of hydrogel with a concentration of 100 mg/ml is applied manually on in the center each backbone, using a light emitting table to guarantee an even spread.

The surface of the 3D printed backbone is roughened with P120 grit sandpaper to improve the adhesion of the hydrogel to the backbone (Figure S7).

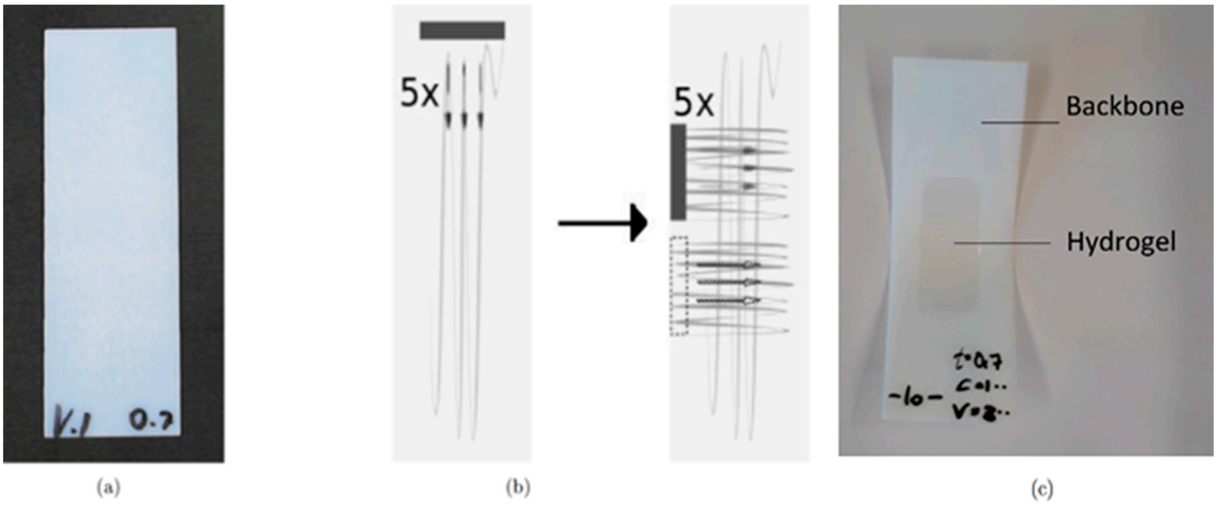

Figure S7. 3D printed SMP backbone for coating with hydrogel. (a) Cleaned and roughened backbone. (b) Grinding scheme. The backbone is roughened five times along the longitudinal axis (left) and five times each on the upper and lower half transverse to the longitudinal axis (right). (c) Hydrogel coated and crosslinked backbone.

Subsequently, the backbones are placed on a heating plate for 45 min at 65°C followed by a drying phase in an oven for another 45 min at 65°C. This procedure dries and thereby shrinks the hydrogel, leading to a bend in the backbone. The dried hydrogel is then cross-linked using an UV-light source with a wavelength of 365 nm (Bio-Link 365, Vilber Lourmat Deutschland GmbH). This process forms a water sensitive hydrogel network that can

actuate the demonstrator when exposed to higher (or different) levels of humidity Klicken oder tippen Sie hier, um Text einzugeben. [36]. The hydrogel coated backbones affixed to foil demonstrators using double-sided adhesive tape (tesa Doppelband Fotostrip, 05338) (Figure S8).

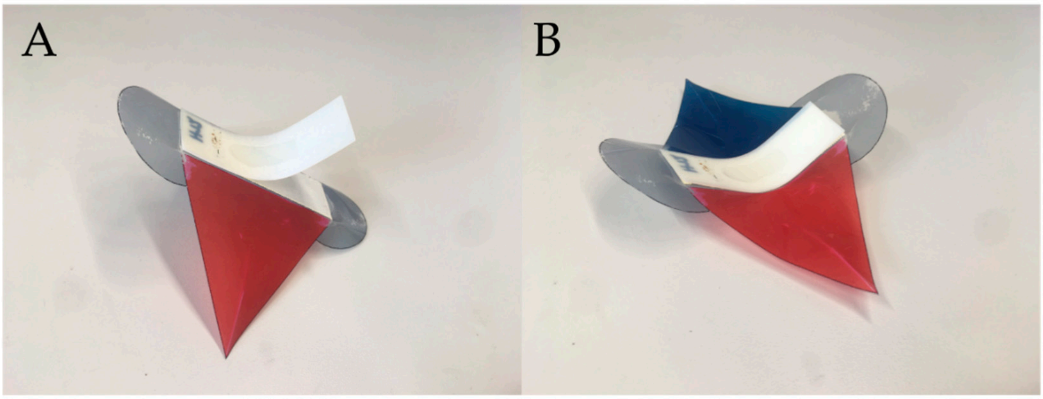

Figure S8. Preparation of full assembly of environmentally triggerable systems stimulus combination system. A cut out piece of adhesive tape is placed on the lower side of the foil demonstrator. The prepared, dried backbone is placed on one side of the of the adhesive tape and then pressed on (A). The demonstrator is in the open, locked position and can be actuated using a water vapor source (B).

To quantify the deformation of the backbone by the hydrogel, the opening angle of the long edges and the slope of the short edges are determined. For this purpose, the curved backbones are placed on a flat platform after drying of the cross-linked hydrogel (Figure S9). Photos of both long and short edges of the backbones are taken with a Lumix DMC-FZ1000 camera, making sure that the respective edge is in the focal plane of the camera and that the reference edge of the pedestal is flat with the camera (Figure S 9).

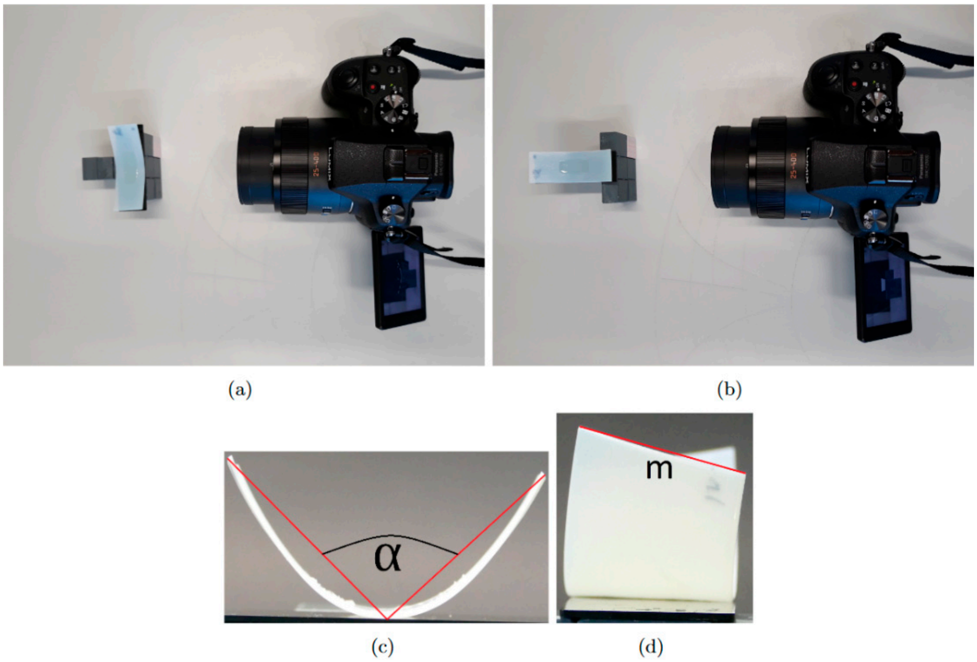

Figure S9. Determination of the opening angles and the torsion of the backbones. (a) Image of the two long edges of a curved backbone. (b) Image capture of the two short edges of a curved backbone. (c) Determination of the angle α. (d) Determination of the slope m of the short edges.

Since the hydrogel is not applied over the full length of the backbone, but only in a certain area in the center, the ends of the backbone remain straight while the center bends. For this reason, instead of calculating the curvature of the backbone, the program ImageJ v1.53a is used to calculate the opening angle α, which is the angle between the support point and the two ends of the backbone (Figure S9). The torsion of the backbone is quantified by the slope m of the short edges (Equation S1), also using the program ImageJ v1.53a.

$$m = \left|\frac{y_1 - y_2}{x_1 - x_2}\right|$$

Equation S1: Calculation of the slope of the short edges of the backbones. m=slope, x,y=coordinates of the corners [mm].

9. Actuation system efficiency calculation

For the calculation of the different actuation systems efficiencies we used the corresponding mean values of the measurements. The equation used for each system are presented below with the corresponding efficiency value. To note here, the efficiency value of the SMA spring system must be considered separately, since a high volume of air must be heated in the climatic chamber, which does not correspond to direct heating, e.g. by Joule heating via electricity, where the efficiencies are significantly higher at up to 60% [37]. *E*= energy consumption, *V*= volume of consumed air.

Pneumatic system:

$$\eta = \frac{E\ (opening) + E(closing)}{1J\ (magnetic\ valves) + V(compressed\ air) * p(system)} = 3{,}7\ \%$$

Magnetic system:

$$\eta = \frac{E(closing) * n(number\ of\ closing\ events)}{E(magnetic\ stirrer)} = 5{,}637\ \%$$

SMA-Spring system:

$$\eta = \frac{E(closing)}{E(climate\ chamber)} = 3{,}3 * 10^{-8}\ \%$$
